# Concurrent self-administered transcranial direct current stimulation and attention bias modification training in binge eating disorder: feasibility randomised sham-controlled trial

**DOI:** 10.1192/bjo.2024.54

**Published:** 2024-06-24

**Authors:** Michaela Flynn, Iain C. Campbell, Ulrike Schmidt

**Affiliations:** Centre for Research in Eating and Weight Disorders, Institute of Psychiatry, Psychology and Neuroscience, King's College London, UK; Centre for Research in Eating and Weight Disorders, Institute of Psychiatry, Psychology and Neuroscience, King's College London, UK; and Outpatient Eating Disorder Service, Maudsley Hospital, South London and Maudsley NHS Foundation Trust, London, UK

**Keywords:** Transcranial direct current stimulation, neuromodulation, attention bias, feeding or eating disorders, binge eating disorder

## Abstract

**Background:**

Binge eating disorder (BED) is a common and disabling condition, typically presenting with multiple psychiatric and obesity-related comorbidities. Evidence-based treatments are either resource-intensive (psychotherapies) or have side-effects (medications): these achieve remission in around 50% of cases. Novel treatments are needed.

**Aims:**

This randomised sham-controlled trial aimed to assess feasibility, acceptability and preliminary efficacy of at-home, self-administered transcranial direct current stimulation (tDCS) and attention bias modification training (ABMT) in adults with binge eating disorder.

**Method:**

Eighty-two participants with binge eating disorder were randomly allocated to real tDCS with ABMT, sham tDCS with ABMT, ABMT only or waitlist control. Intervention groups received ten sessions of their allocated treatment over 2–3 weeks. tDCS (2 mA, 20 min) was self-administered using a bilateral (anode right/cathode left) montage targeting the dorsolateral prefrontal cortex. Outcomes were assessed at baseline, post-treatment and 6-week follow-up.

**Results:**

Prespecified feasibility criteria (recruitment ≥80 participants and retention rate ≥75%) were exceeded, and treatment completion rates were high (98.7%). All interventions reduced binge eating episodes, eating disorder symptoms and related psychopathology between baseline and follow-up, relative to waitlist control (medium-to-large between-group effect sizes for change scores). Small-to-medium effect sizes for change scores favoured real tDCS with ABMT versus comparators, suggesting the verum intervention produces superior outcomes.

**Conclusions:**

At-home, self-administered tDCS with ABMT is feasible and acceptable, and preliminary data on efficacy are promising. This approach could be a useful and scalable alternative or adjunct to established treatments for binge eating disorder. Confirmatory trials can, and should, be pursued.

Binge eating disorder (BED) is a distressing and disabling eating disorder characterised by recurrent episodes of binge eating during which the individual consumes objectively large amounts of food and experiences a sense of loss of control.^1^ Lifetime prevalence rates (pooled for gender) can be up to 4.7%, and rates are elevated in minoritised groups and those affected by food insecurity.^2^ Comorbidity with mood, anxiety or impulse control disorders; attention-deficit hyperactivity disorder; or obesity and associated physical health problems is common.^2^ Psychological therapies, particularly cognitive–behavioural therapy, are recommended as a first-line intervention: these improve mood but only enable around 50% of people to abstain from binge eating.^2^ Pharmacotherapy is limited, with only lisdexamphetamine approved for use in BED. This reduces binge eating episodes and promotes weight loss; however, side-effects are common, and long-term follow-up data are lacking.^2^ Thus, there is a need for novel, accessible and scalable treatment options. These may achieve better outcomes if they take a brain-directed approach.

## Neurocognitive targets for treatment

Impulsivity, which is characterised by reduced inhibitory control and increased reward sensitivity, may have a role in perpetuating binge eating behaviour in BED.^3^ This fits with the core psychopathology of BED and with reports that BED is highly comorbid with impulse control disorders. Individuals with BED score highly on measures of impulsivity, and there is evidence that BED is associated with reduced inhibitory control in the context of food and increased sensitivity to food-related rewards.^3^ Studies have also reported attention biases toward food and difficulties disengaging from these cues.^4,5^ This contributes to food craving, a precipitant of binge eating in BED. These features of BED have been associated with distinct activation patterns in prefrontal and striatal regions.^6^ Specifically, neuroimaging studies comparing participants with BED to lean and body mass index (BMI)-matched controls report that those with BED show hypoactivation in the dorsolateral prefrontal cortex (dlPFC)^7–10^ and medial prefrontal cortiex^7,11^ during food-related tasks probing inhibitory control^7,8,11^ and attention bias.^9,10^ During food cue exposure, they also show increased brain activity in reward processing regions (e.g. ventral striatum and orbitofrontal cortex).^11–13^ This suggests that BED is underpinned by altered recruitment of networks involved in self-regulation and reward processing. Thus, interventions that restore typical functioning to these circuits and reduce attention biases toward food, may provide new treatment options for BED.

## Attention bias modification training

Functional restoration may be achieved through attention bias modification training (ABMT), a neurocognitive training method that aims to modify the preferential allocation of attention toward disorder-relevant stimuli.^14^ ABMT was developed by modifying the dot-probe task for assessing attention bias,^15^ however, studies suggest that training based on modified inhibitory control paradigms, such as the anti-saccade task, may have more potent effects when applied to appetitive stimuli, such as food.^16,17^ In BED and obesity, ABMT aims to alter automatic attention biases toward food cues by training attention away from high-calorie food cues and/or toward healthy food cues. This attentional shift is thought to implicitly modify the valence of food cues (i.e. high-calorie food will become less rewarding and/or healthy food more appealing), and subsequently alter eating behaviour. Although findings have been mixed, meta-analyses have shown that ABMT reduces high-calorie food consumption and craving in adults with ‘healthy’ weight^18^ and adults with overweight or obesity.^19^ Few studies have assessed the effects of ABMT in BED, but preliminary findings are promising. A single-session, randomised controlled trial (RCT) (*n* = 47) reported significant reductions in subjective food craving after participants with BED were trained to look away from food cues using ABMT.^20^ Second, an open feasibility study (*n* = 9) involving three sessions of ABMT per week for 8 weeks reported that weight, eating disorder symptoms, binge eating episodes and attention bias toward food were reduced post-treatment and at 3-month follow-up, relative to baseline.^21^ Third, a feasibility study compared ABMT and mindfulness training (eight laboratory-based sessions over 8 weeks, plus daily at-home training) in adults with obesity with or without BED (*n* = 45). It showed that, compared with waitlist controls, ABMT was associated with fewer binge eating episodes, reduced BMI and less severe eating disorder psychopathology at 4 weeks post-treatment, and that the effect of treatment on eating disorder psychopathology was greater in the ABMT arm compared with mindfulness training.^22^ Thus, ABMT may be a useful, low-cost, scalable tool for treating BED: there is, however, a need to investigate whether combining ABMT with extant or novel treatments enhances efficacy.

## Transcranial direct current stimulation

Transcranial direct current stimulation (tDCS) is a neuromodulation technique that may be used to improve self-regulatory processes in BED, and preliminary studies indicate its therapeutic potential.^23^ In tDCS, a constant, weak (1–3 mA) direct current is applied via electrodes placed on the scalp to increase or decrease neuronal excitability in regions beneath the electrodes and in functionally connected networks. These changes in excitability outlast the stimulation period (up to 60 min after a single session) and, with repeated administration, may lead to lasting changes in brain function.^24^ tDCS is safe, well tolerated, inexpensive and scalable,^24^ particularly because of the availability of devices designed for at-home self-administration. A meta-analysis of studies suggested that a single session of tDCS targeting the dlPFC was associated with small-to-moderate reductions in food craving; however, this should be interpreted with some consideration of the methodological heterogeneity in the studies. The studies were conducted in groups with varying degrees of food craving (e.g. samples include healthy adults that reported strong craving for food, adults with obesity, adults with bulimia nervosa and adults with BED) and they used different tDCS protocols.^25^ In BED specifically, proof-of-concept studies suggest that a single session of 2 mA tDCS targeting the dlPFC, using either a bilateral (anode right/cathode left) montage^26^ or right anodal montage,^27^ reduces food cravings, desire to binge eat and difficulties with inhibitory control in the context of food (small-to-moderate effect sizes).

The effect of tDCS on craving may be amplified when multiple sessions are delivered. Indeed, meta-analyses of studies in substance use disorders suggest reductions to craving are greatest when tDCS sessions are repeated, bilateral stimulation is used, the anodal electrode is placed on the right dlPFC, current intensities range from 1.5 to 2 mA and stimulation sessions last 20 min.^28^ Two sham-controlled RCTs have examined the effect of multiple sessions of tDCS targeting the dlPFC in adults with BED. The first (*n* = 40) assessed the effect of ten sessions of 2 mA bilateral tDCS (20 mins; anode left/cathode right) on attention bias toward food, food craving and cognitive flexibility.^29^ At post-treatment and 6-week follow-up, real tDCS (versus sham) was associated with significant reductions in attention bias toward food and food craving, although effect sizes were small. The second trial (*n* = 41) assessed the feasibility and clinical effects of six sessions of laboratory-based 2 mA anodal tDCS (20 mins; anode right dlPFC/reference left deltoid) and concurrent food-specific inhibitory control training.^30^ Findings suggest that training enhanced by tDCS is associated with substantial and sustained reductions in binge eating episodes. Specifically, at 4 weeks post-treatment, both groups reported substantial reductions in monthly binge eating episodes relative to baseline; however, at 3 months post-treatment, those who received training together with real tDCS reported significantly fewer monthly episodes of binge eating than those who received sham tDCS.

Neural responses to brain stimulation are likely to depend on physiological and cognitive states during stimulation,^31^ i.e. tDCS preferentially modulates neural networks that are active during stimulation. Indeed, when frontal brain regions involved in cognitive control are stimulated, studies report network-wide effects that are dependent on the locus of attention.^31^ As such, exposure to or engagement with disorder-relevant stimuli (e.g. food) or tasks during tDCS may promote targeted restoration of neural functioning, which may translate to clinically meaningful behaviour change. Thus, an ‘online’ approach, where tDCS is combined with ABMT, may optimise both tDCS and ABMT treatments.

Questions remain regarding optimal parameters, but it is generally accepted that multiple sessions in close succession (e.g. daily) are required to achieve lasting therapeutic effects. Therefore, laboratory-based tDCS interventions require a significant time and travel commitment by participants. Home-based tDCS is an accessible and scalable alternative to laboratory-based tDCS. Evidence from RCTs in other psychiatric disorders indicates that at-home self-administered tDCS is feasible, acceptable and safe, particularly when real-time videoconferencing is used to monitor treatment fidelity, comprehensive training is provided and specialist equipment is used to reduce the risk of electrode misplacement.^32^ To our knowledge, the feasibility and acceptability of at-home self-administered tDCS has yet to be evaluated in BED.

## The present study

This trial assessed the feasibility and acceptability of ten sessions (over 2 consecutive weeks) of at-home self-administered bilateral tDCS of the dlPFC (2 mA, anode right/cathode left, 20 min) together with ABMT in adults with BED. A further aim was to obtain preliminary data on clinical efficacy to inform the basis for a future confirmatory trial.

## Method

This trial was named TANDEM. Details of trial design, participants and procedures have been reported previously.^33,34^ The trial was registered with ClinicalTrials.gov (trial identifier: NCT04424745). All procedures contributing to this work comply with the ethical standards of the relevant national and institutional committees on human experimentation and with the Helsinki Declaration of 1975, as revised in 2008. All procedures involving human participants were approved by the London and Fulham NHS research ethics committee (reference 20/LO/0936). Written informed consent was obtained from all participants.

### Design, participants and setting

TANDEM was a single-blind, randomised, sham-controlled feasibility trial with four parallel arms: (a) real tDCS plus ABMT, (b) sham tDCS plus ABMT, (c) ABMT only and (d) waitlist control. Outcomes were assessed at baseline (time point 0), post-treatment (time point 1; immediately after treatment completion, or 2 weeks post-randomisation for waitlist control) and follow-up (time point 2; 6 weeks after end of treatment, or 8 weeks post-randomisation for waitlist control).

The predetermined sample size target was 80 participants (20 per group). Participants were recruited between 1 March 2021 and 28 February 2022. Participants were right-handed adults (≥18 years old) who were overweight or obese (BMI ≥25 kg/m^2^) and met the DSM-5 criteria for BED diagnosis.^1^ Participants had normal or corrected-to-normal vision, and access to a computer with a webcam. Main exclusion criteria were contraindications to tDCS (e.g. seizures or migraines). Participants were recruited from the community via online advertisements and from the South London and Maudsley (SLaM) NHS Foundation Trust's eating disorder service.

### Intervention

Participants received ten sessions of tele-supervised treatment over 2–3 weeks (i.e. one session per week day until ten were completed). Sessions involved either ABMT with real/sham tDCS, or ABMT only. ABMT was completed on a laptop or desktop computer and lasted 10–15 min. During ABMT, participants were trained to ‘look toward’ low-calorie food cues and ‘look away’ from high-calorie food cues, using a modified version of the anti-saccade task.^17^ tDCS was delivered using specialist equipment designed for at-home self-administration (the HDC Kit with MindCap™ by Newronika). In the real tDCS condition, stimulation was delivered with the anode over the right dlPFC and the cathode over the left dlPFC, at an intensity of 2 mA for 20 min. In sham tDCS, participants set up electrodes in the same way and received active stimulation for 60 s at the start and end of the session. Participants started stimulation 5 min before beginning ABMT, so that stimulation and ABMT concluded approximately simultaneously. Further details about the equipment and intervention are provided in the Supplementary Material available at https://doi.org/10.1192/bjo.2024.54.

### Outcomes

Prespecified criteria for feasibility were recruitment of at least 80 participants and retention to follow-up (time point 2) rates of ≥75%. Blinding success was assessed using a binary (real/sham) question, and was considered successful if participants correctly guessed their allocation at a rate comparable to chance.

Acceptability was assessed in two ways. First, two binary (yes/no) questions asked whether the participant would continue the intervention if they could, and whether they would, recommend the intervention to a friend with BED. Endorsement rates ≥75% would indicate that the intervention was acceptable. Second, after each treatment session, participants who received real/sham tDCS completed a ten-point visual analogue scale of tDCS-related discomfort. A group average rating of ≤4 (i.e. mild discomfort) would indicate that the intervention was well tolerated. Frequency and severity of side-effects were also reported.

The primary clinical outcome was change in number of monthly objective binge episodes (OBEs) from baseline to time point 2. Secondary clinical outcomes related to the change in eating disorder symptoms, general psychopathology, food craving, difficulties with emotion regulation and eating disorder-related quality of life from time point 0 to time points 1 and 2.

### Clinical outcome measures

The Eating Disorder Examination Questionnaire (EDE-Q)^35^ was used to confirm BED diagnosis and assess eating disorder symptom severity, the number of monthly OBEs in the previous 28 days and BMI. General psychopathology was assessed with the Depression, Anxiety and Stress Scale (21-item version; DASS-21).^36^ The Food Craving Questionnaire (trait version; FCQ-T),^37^ Difficulties in Emotion Regulation Scale (DERS)^38^ and Clinical Impairment Assessment (CIA)^39^ were also administered. For all measures, higher scores indicated greater levels of symptom severity or impairment.

### Procedure

Potential participants were screened by telephone against inclusion/exclusion criteria. Written informed consent was obtained from all participants. Eligible participants completed the baseline assessment, after which they were randomised to one of the trial arms. Intervention groups completed ten sessions of their allocated treatment over 2–3 weeks. Waitlist participants received no experimental treatment during this time. Participants completed the time point 1 assessment 1–3 days after the final session of treatment or 2 weeks post-randomisation (waitlist group), and the time point 2 assessment 6 weeks after completing treatment or 8 weeks post-randomisation. Waitlist control participants were then invited to start ABMT. Participants completed all components of treatment and assessment from home with researcher support via video call.

At all three assessments, participants completed questionnaire measures and neuropsychological tasks. Data relating to neuropsychological tasks and process outcomes will be reported elsewhere.

### Data analysis

Analyses were completed in the intent-to-treat population, using RStudio (version 2023.03.01 for Windows by Posit; see https://posit.co/download/rstudio-desktop/). Descriptive statistics were used to assess recruitment and retention rates, and intervention acceptability ratings. Effect sizes (Cohen's *d*) for change scores were used to examine the effect of treatment on clinical symptoms (*d* ≤ 0.2 are small, *d* ≤ 0.5 are moderate and *d* ≤ 0.8 are large). The effect of treatment on monthly OBEs was modelled with a generalised linear model, with a negative binomial distribution and log link. In our protocol,^33^ we indicated that this relationship would be modelled with a Poisson distribution; however, due to overdispersion, the negative binomial distribution provided superior model fit.^40^ Given limited power, negative binomial regression and *P*-values are reported for exploratory purposes only.

## Results

### Feasibility outcomes

#### Participant flow, attendance and retention

**Fig. 1:**
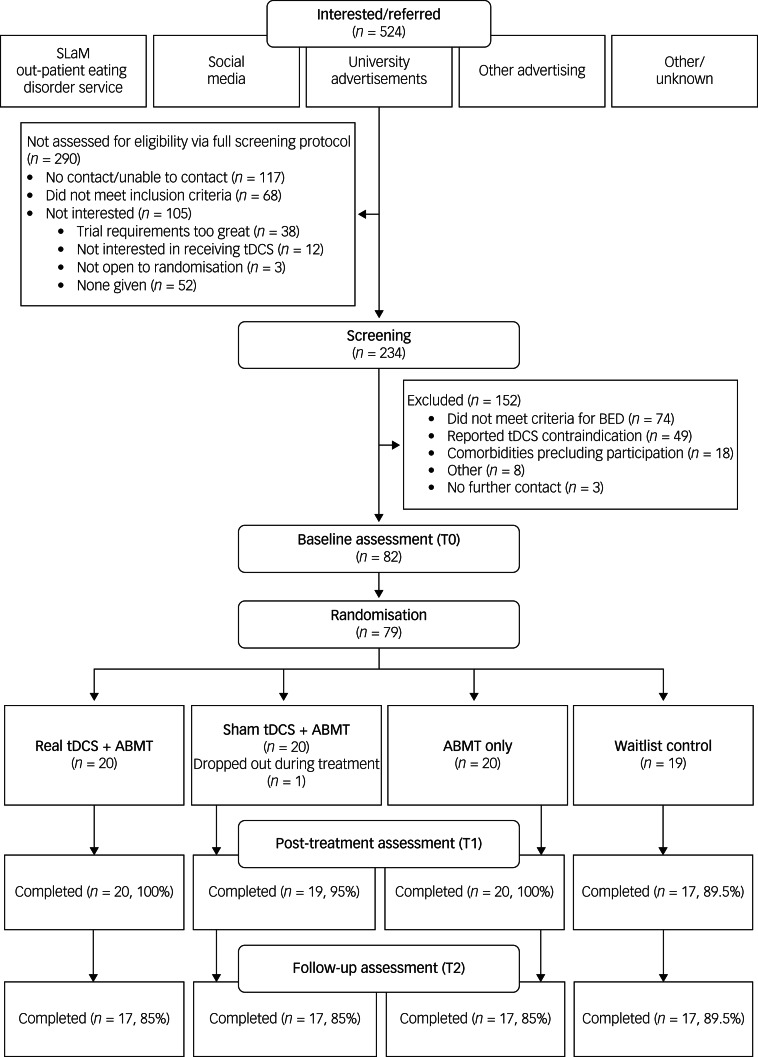
Participant flow. ABMT, attention bias modification training; BED, binge eating disorder; SLaM, South London and Maudsley NHS Foundation Trust; tDCS, transcranial direct current stimulation.

Eighty-two participants completed baseline assessment. Before randomisation, three withdrew, citing a positive COVID-19 test as the reason. Seventy-nine participants were randomised to one of the four study arms: real tDCS plus ABMT (*n* = 20), sham tDCS plus ABMT (*n* = 20), ABMT only (*n* = 20) and waitlist control (*n* = 19) (see Fig. 1 for a participant flow diagram). During treatment, one participant (sham tDCS plus ABMT) tested positive for COVID-19 and stopped after five sessions. All others completed treatment. Seventy-six completed the time point 1 assessment (92.7%), and 68 completed the time point 2 assessment (82.8%). Independent *t*-tests revealed no significant differences between those who completed the time point 1 and 2 assessments and those who did not, based on age, BMI, EDE-Q global score or DASS-21 total score. Missing data were managed with case-wise deletion. Intervention acceptability and blinding success are reported in the Supplementary Material.

#### Participants

Baseline clinical characteristics and demographics are presented in Table 1 and the Supplementary Material.

**Table 1:** Baseline characteristics

	Whole sample	Real tDCS + ABMT	Sham tDCS + ABMT	ABMT only	Waitlist control
	(*N* = 82)	(*n* = 20)	(*n* = 20)	(*n* = 20)	(*n* = 20)
Demographic details					
Age, years, mean (s.d.)	42.18 (9.49)	40.40 (9.83)	40.45 (7.88)	43.70 (10.60)	43.52 (9.85)
Female gender, *n* (%)	80 (97.60)	19 (95.0)	19 (95.0)	20 (100.00)	19 (100.00)
Clinical characteristics					
BMI, kg/m^2^, mean (s.d.)	39.96 (7.46)	39.00 (6.79)	39.18 (8.47)	40.48 (8.61)	40.20 (6.58)
History of bariatric surgery, *n* (%)	3 (3.7)	1 (0.05)	1 (0.05)	1 (0.05)	0 (0.00)
EDE-Q Global, mean (s.d.)	4.04 (0.86)	4.00 (0.80)	3.98 (0.79)	4.20 (0.80)	3.98 (1.11)
EDE-Q monthly OBEs, mean (s.d.)	19.63 (9.46)	19.58 (10.20)	18.85 (11.90)	21.05 (8.81)	19.00 (6.65)
DASS-21 Depression, mean (s.d.)	16.39 (11.04)	18.10 (11.54)	14.30 (10.02)	17.80 (11.66)	15.89 (12.06)
DASS-21 Anxiety, mean (s.d.)	8.02 (6.69)	7.60 (6.79)	8.20 (8.26)	9.00 (6.73)	7.26 (5.04)
DASS-21 Stress, mean (s.d.)	17.41 (8.11)	18.70 (10.63)	16.60 (7.37)	18.50 (7.13)	16.63 (7.43)
DASS-21 total, mean (s.d.)	41.83 (21.79)	44.40 (25.25)	39.10 (21.75)	45.30 (20.10)	39.79 (21.86)
FCQ-Tr total, mean (s.d.)	63.05 (8.25)	63.90 (8.40)	60.45 (7.80)	64.30 (8.66)	62.79 (8.54)
DERS, mean (s.d.)	32.23 (15.95)	36.40 (18.41)	29.90 (14.89)	32.60 (14.67)	30.00 (16.75)
CIA, mean (s.d.)	1.53 (0.56)	1.49 (0.59)	1.43 (0.40)	1.65 (0.61)	1.50 (0.64)
Comorbidities, *n* (%)					
Depression	50 (61.00)	15 (75.00)	11 (55.00)	11 (55.00)	12 (63.20)
Anxiety	34 (41.50)	9 (45.00)	9 (45.00)	8 (40.00)	8 (42.11)
Diabetes mellitus type 2	13 (15.90)	6 (30.00)	2 (10.00)	3 (15.00)	2 (10.53)
Prediabetes	7 (8.50)	1 (0.05)	1 (0.05)	3 (15.00)	1 (0.05)
Hypertension	24 (29.30)	5 (25.00)	7 (35.00)	8 (40.00)	2 (10.53)
Hyperlipidaemia	15 (18.30)	1 (0.05)	5 (25.00)	6 (30.00)	2 (10.00)
Hypothyroidism	13 (15.90)	4 (20.00)	2 (10.00)	3 (15.00)	3 (15.79)
Concurrent treatment, *n* (%)					
Guided self-help	3 (3.66)	1 (0.05)	1 (0.05)	1 (0.05)	0 (0.00)
Antidepressant medication	41 (50.00)	11 (55.00)	10 (50.00)	9 (45.00)	10 (52.63)

No statistically significant difference between groups at the *P* < 0.05 level. tDCS, transcranial direct current stimulation; ABMT, attention bias modification training; BMI, body mass index; EDE-Q; Eating Disorders Examination Questionnaire; OBEs, objective binge eating episodes; DASS-21, Depression, Anxiety and Stress Scale, 21 items; FCQ-Tr, Food Craving Questionnaire-Trait reduced version; DERS, Difficulties with Emotion Regulation Scale; CIA, Clinical Impairment Assessment.

#### Intervention acceptability

At time points 1 and 2, 100% of participants who received real and sham tDCS with ABMT declared that they would recommend the intervention to others, and most who received real tDCS indicated that they would continue treatment if they could (time point 1: 100%; time point 2: 95%). Participants who received sham tDCS were less likely to endorse continuing treatment (time point 1: 74%; time point 2: 68.4%). Most who received ABMT only indicated that they would recommend treatment to a friend with BED (time point 1: 76%; time point 2: 72%), however, few indicated that they would elect to continue treatment (time point 1: 46%; time point 2: 23%).

#### tDCS tolerability

The real tDCS plus ABMT participants reported mild discomfort during stimulation (mean rating: 1.8/10, s.d. = 1.22). Sham tDCS plus ABMT participants reported negligible tDCS-related discomfort (mean rating: 0.46, s.d. = 0.34). Both groups reported few tDCS-related side-effects, and in all cases, side-effects were mild; two real tDCS plus ABMT participants and two sham tDCS plus ABMT participants reported headache on one occasion, and one sham tDCS plus ABMT participant reported mild neck pain after session one. Incidence of side-effects did not differ between groups (*Χ*^2^(1) = 0.46, *P* = 0.638).

#### Clinical outcomes

Outcomes relating to OBEs (primary outcome) and mood are reported here. Between-group effect sizes for change scores to time point 1 and time point 2, relative to time point 0, mean scores for clinical outcome measures at time points 1 and 2, and findings relating to other secondary clinical outcomes are reported in the Supplementary Material.

#### Episodes of objective binge eating

**Fig. 2:**
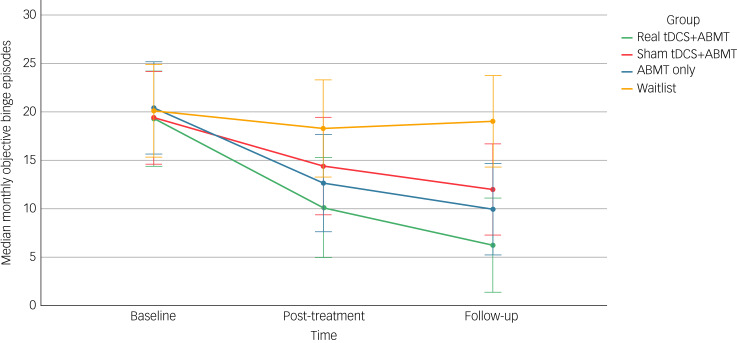
Median monthly objective binge episodes from baseline to follow-up, with 95% confidence intervals. ABMT, attention bias modification training; tDCS, transcranial direct current stimulation.

All intervention groups reported a reduction in OBEs from time point 0 to time point 2 (Fig. 2). When comparing each intervention group with the waitlist control, effect sizes for change scores to follow-up were large, and favoured the intervention group (real tDCS plus ABMT: *d =* −1.33, 95% CI −2.08 to −0.56*;* sham tDCS plus ABMT: *d =* −1.21, 95% CI −1.93 to −0.46; ABMT only: *d* = −1.05, 95% CI −1.77 to −0.33). When comparing the real tDCS plus ABMT group with comparison intervention groups, small-to-moderate effect sizes for change scores, which favoured the real tDCS plus ABMT group, were observed (sham tDCS plus ABMT: *d =* −0.58, 95% CI −1.28 to 0.12; ABMT only: *d* = −0.21, 95% CI −0.90 to 0.47).

Negative binomial regression indicated that, overall, treatment group was a significant predictor of the number of monthly OBEs reported at time point 2 (*Χ*^2^ = 9.55, d.f. = 3, *P* < 0.05). Based on this model, the predicted incidence rate for OBEs at time point 2 was 33% lower in the real tDCS plus ABMT group than in the waitlist group (exp(*β*) = 0.33, s.e. = 0.37, *P* < 0.01, 95% CI −0.16 to −0.67). The predicted incidence rate for OBEs following sham tDCS plus ABMT and ABMT only were not significantly different from the waitlist control (*P* = 0.19 and *P* = 0.07, respectively) or from real tDCS plus ABMT (*P* = 0.54 and *P* = 0.31, respectively).

#### Mood

**Fig. 3:**
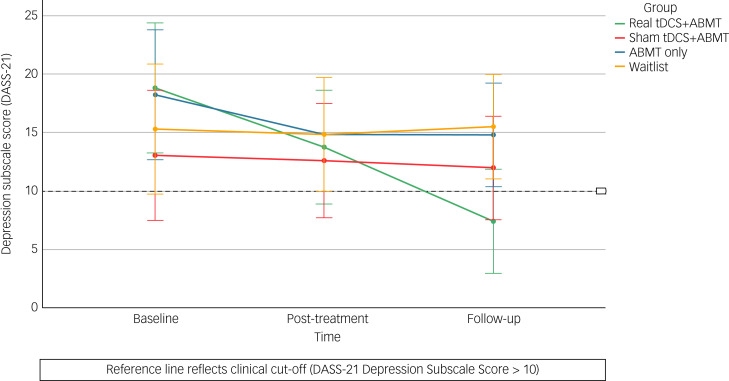
Mean scores on the depression subscale (DASS-21) from baseline to follow-up, with 95% confidence intervals. ABMT, attention bias modification training; DASS-21, Depression, Anxiety and Stress Scale, 21 items; tDCS, transcranial direct current stimulation.

Scores on the DASS-21 depression subscale were reduced in all intervention groups between time point 0 and time point 2 (Fig. 3). When comparing each intervention group with the waitlist control, effect sizes for change scores to follow-up were large, and favoured the intervention group (real tDCS plus ABMT: *d =* −1.20, 95% CI −1.92 to −0.46*;* sham tDCS plus ABMT: *d =* −0.19, 95% CI −0.87 to 0.48; ABMT only: *d* = −0.62, 95% CI −1.31 to 0.07). When comparing the real tDCS plus ABMT group to comparison groups, large effect sizes for change scores favoured the real tDCS plus ABMT group (sham tDCS plus ABMT: *d =* −1.06, 95% CI −1.77 to −0.33; ABMT only: *d* = −0.86, 95% CI −1.56 to −0.15).

## Discussion

Our findings demonstrate good feasibility and acceptability of the intervention, and support pursuit of a confirmatory trial. Recruitment rates exceeded prespecified criteria for feasibility, and retention to follow-up rates were high. Treatment session attendance was excellent: 78 out of 79 participants completed all ten treatment sessions within the 3-week maximum time frame. tDCS with ABMT was well tolerated: mean discomfort ratings were low, and side-effects were few and infrequent. Participant ratings indicated that tDCS with ABMT was highly acceptable. In fact, acceptability ratings were higher at both time points for the concurrent treatment than for ABMT only. Similarly, rates for treatment adherence and retention to follow-up compare favourably with related trials, particularly those involving ABMT only. For example, in an open-label study of ABMT in BED, Boutelle et al^21^ reported that 60% of participants completed treatment (one in-person and two at-home ABMT sessions per week, for 8 weeks), and in their trial comparing ABMT with mindfulness training in adults with obesity, Mercado et al^22^ reported that participants completed 35% of the recommended at-home ABMT sessions (six sessions per week, for 8 weeks). The reason for superior ABMT adherence in TANDEM is unclear. It could reflect greater treatment acceptability because ABMT was being used as an adjunct to tDCS. Alternatively, it could be because of the supervision/monitoring the participants received, or it may be related in some way to the cohort or the time during which the trial was carried out (i.e. the COVID-19 pandemic).

Preliminary findings regarding efficacy are promising. At the 6-week follow-up, monthly OBEs were reduced in all intervention groups; however, the reduction was greatest in those who received real tDCS. This is in accordance with a feasibility RCT in BED using a comparable intervention. In a sham-controlled trial of six sessions of laboratory-based tDCS applied to the dlPFC (2 mA, 20 min, anode right/reference left deltoid) with inhibitory control training, Giel et al^30^ reported no significant difference between real and sham groups for BED symptoms at 4 weeks post-treatment; however, significant differences between groups were observed at the 3-month follow-up, with those who received real tDCS with inhibitory control training reporting significantly fewer episodes of binge eating than those who received training with sham tDCS. This suggests that the synergistic effects of tDCS and training take time to emerge, and may be related to the strengthening of tDCS-related effects over time. It may also be that tools assessing OBEs ask about the previous 4 weeks, so changes to eating behaviour may take weeks to be apparent. In Giel et al,^30^ training was also based on an anti-saccade task, but participants were not cued to perform an anti-saccadic or pro-saccadic response (as in the TANDEM trial, i.e. by presentation of a red or blue dot before food cue presentation). Rather, high-calorie food pictures were presented in their peripheral vision immediately after the presentation of a fixation cross, and participants were directed to perform the anti-saccadic response only. It is possible that training protocol differences meant that different mechanisms were targeted, but it is also possible that there is a shared mechanism of action, and this may explain comparable intervention outcomes.

The intervention was also associated with improvement across several other clinical domains. All intervention groups reported improvement in mood, and improvement was most pronounced in those who received real tDCS with ABMT. In this group, we observed a linear reduction in symptoms of low mood between baseline and follow-up indicative of an antidepressant effect. Studies have shown that tDCS targeting the dlPFC has moderate-to-strong antidepressant effects in major depression, particularly when depression is acute (as opposed to treatment resistant) and when tDCS is combined with selective serotonin reuptake inhibitors (SSRIs),^41^ although these studies typically use a bilateral (anode left/cathode right) or anodal (anode left/extracephalic right) montage. Half of our participants reported concurrent use of antidepressant medication (most commonly SSRIs), and mean scores on the depression subscale at baseline indicated that, on average, symptoms of low mood were moderately severe. The definite mechanism of tDCS is not well understood, and it is possible that improvement in mood was related to tDCS itself. However, it may also be a consequence of the amelioration of BED symptoms, or related to other factors, such as the therapeutic alliance with the technician overseeing tDCS administration.

Participants in the real tDCS with ABMT group also reported greater weight loss between baseline and follow-up (mean_Δ_ = −1.28 kg/m^2^) than ABMT with sham (mean_Δ_ = −0.52 kg/m^2^) and ABMT only (mean_Δ_ = −0.07 kg/m^2^). This suggests substantial and sustained changes to eating behaviour in the real tDCS with ABMT group. This may be driven by the changes to day-to-day life linked to improvement in mood (e.g. greater participation in physical activity or reduced stress), fewer episodes of binge eating or reduced craving for food. Future studies should measure food consumption as well as craving for food, to clarify the mechanism driving weight loss.

All groups reported fewer eating disorder symptoms and reduced craving for food at follow-up, and there was greater change in the group who received real tDCS with ABMT. This is consistent with reports that tDCS reduces food craving in the short term,^26,27^ and indicates that craving reduces further post-treatment. However, EDE-Q scores were only marginally above the clinical cut-off at baseline, despite full-syndrome BED, moderate-to-severe distress (i.e. moderate-to-high scores on the DASS-21) and high rates of psychiatric and obesity-related comorbidity (e.g. 61.0% reported depression, 50% reported hypertension and 24.4% reported diabetes or prediabetes). Semi-structured clinical interviews may be more sensitive to change in eating disorder psychopathology in BED, and should be considered in future studies.

To the best of our knowledge, this is the first study to deliver at-home tDCS to adults with an eating disorder. It shows that adults with eating disorders view this approach to be credible, accessible and worth trying, so research exploring how these tools may be leveraged to improve outcomes for people with eating and weight disorders is encouraged. In view of comparable therapeutic effects following a multisession intervention using anodal tDCS of the right dlPFC with inhibitory control training in adults with BED, there is a need for research comparing unilateral and bilateral tDCS protocols. At-home self-administered tDCS overcomes a number of barriers to neuromodulation as a treatment (e.g. time and travel commitments), and BED treatment in general (e.g. less resource intensive than psychotherapy). However, even when equipment is designed for self-administration at home, it cannot be guaranteed that stimulation has been delivered precisely to the correct cortical target, or that it has been delivered exactly the same way each time. Equipment that can be tailored to the individual may improve the precision of home-based tDCS. There is also a need to increase knowledge on how tDCS affects brain functioning, how it augments other treatments and the parameters most important in terms of treatment response. This will necessitate examining how therapeutic effects differ when parameters such as dose, intensity, current flow, target and adjunct versus no adjunct are changed. Additionally, studies that identify individual biological, cognitive and clinical markers of intervention response are needed so that optimal parameters may be selected on an individual basis.

Further research clarifying the efficacy, specificity and mechanisms of ABMT for BED is warranted. We observed a potent effect of ABMT on binge eating, and effect sizes are larger than previously reported. This suggests the anti-saccade paradigm used may target the neurocognitive maintenance mechanisms of BED more effectively than the dot-probe paradigm. Indeed, one study has argued that cognitive training that targets inhibitory control may be more suitable for BED than traditional approaches to ABMT (i.e. dot-probe paradigms), and that the anti-saccade paradigm may be the optimal paradigm for influencing food-related disinhibition.^16^ In the anti-saccade task, anti-saccadic responses are viewed to demonstrate inhibitory control. Thus, training an individual to perform anti-saccadic responses in the context of food may improve inhibitory control in the context of food. However, in our paradigm, participants were cued to perform an anti-saccadic response. As such, they may have practiced the suppression of an automatic orientation response to food, rather than an inhibitory response to food. Consideration for the change mechanisms necessitates that we measure information processing during training administration by using instruments able to differentiate between attention-related and inhibitory control processes (e.g. laboratory-based eye tracking or neuroimaging). Alternatively, better outcomes may be explained by the one-to-one supervision provided throughout treatment, i.e. regular contact with a supportive supervisor may introduce a therapeutic alliance or improve treatment adherence.

It should also be noted that other approaches to neurocognitive training (e.g. emotion regulation training) have shown promise in BED. Thus, it is possible that these will produce equivalent or superior outcomes when delivered with concurrent tDCS, or that different approaches may be better suited to different individuals.

The study has some limitations. The trial took place during the COVID-19 pandemic, when people were spending more time at home and interest in virtually delivered psychological care was high. This may have positively influenced attitudes toward at-home self-administered tDCS with ABMT, as well as willingness to participate. Additionally, as all trial elements were completed remotely, we were able to recruit widely. This likely contributed to the geographic and demographic diversity of our sample, although male representation was low (*n* = 2). Adoption of a fully remote design required that we sacrifice control over factors that might introduce unwanted variability or bias during outcome assessment. Reliance on self-report data, particularly for our primary outcome measure (binge eating episodes as measured by the EDE-Q), may have led to under- or overreporting of BED symptoms and related psychopathology. Future trials could consider hybrid designs that benefit from the rigour of laboratory-based assessment and clinician-led measurement of BED pathology, but retain a remote treatment model. Longer follow-up (e.g. 12 months post-treatment) is also encouraged. The COVID-19 pandemic also prompted our pragmatic decision to use a single-blind study design. Participant blinding to tDCS real/sham allocation was effective; however, as personnel delivering treatment were not blind to treatment allocation, there is some possibility that experimenter bias influenced our findings.

In conclusion, we investigated the feasibility of at-home self-administered tDCS in eating disorders, and our findings indicate that this may be a welcome new avenue for treatment. This is particularly relevant for BED, a patient group that is underserved, complex and stigmatised. Our findings indicate that home-based tDCS with ABMT is a feasible and acceptable intervention for adults with BED, and data on clinical efficacy are promising; all interventions produced improvements in core BED symptoms and related psychopathology, and there was evidence that real tDCS with ABMT produces superior treatment outcomes. As such, this approach could be a useful and scalable alternative or adjunct to established treatments. Future studies of home-based tDCS with ABMT, or other suitable adjuncts, are encouraged.

## Supporting information

Flynn et al. supplementary material 1 Flynn et al. supplementary material

Flynn et al. supplementary material 2 Flynn et al. supplementary material

Flynn et al. supplementary material 3 Flynn et al. supplementary material

## Data Availability

Anonymised data are attached to this publication in the Supplementary Material. Data provided include sufficient information for an independent researcher to reproduce the reported methodology.
