# Supplementary material for: Concurrent self-administered transcranial direct current stimulation and attention bias modification training in binge eating disorder: feasibility randomised sham-controlled trial
Source: BJPsych Open. 2024 Jun 24;10(4):e118. doi: 10.1192/bjo.2024.54 (PMC11363089; doi:10.1192/bjo.2024.54)
Supplement: Flynn et al. supplementary material 1 — Flynn et al. supplementary material [file S2056472424000541sup001.docx]

# Supplementary Materials

## Supplementary Methods

### Randomisation and blinding procedure

Sealed EnvelopeTM randomised participants at a ratio of 1:1:1:1 using a restricted randomisation algorithm which stratified by age, BMI, and gender. Only participants were blind to real/sham tDCS allocation. Participants were unblinded after the T2 assessment. Those who received ABMT with sham tDCS were not offered any additional treatment.

### Intervention Description

Participants completed 10 sessions of tele-supervised treatment over 2-3 weeks (i.e., week daily sessions until 10 sessions were completed). Sessions involved either concurrent ABMT and real/sham tDCS, or ABMT only.

Attention Bias Modification Training (ABMT)

ABMT was developed by modifying the dot-probe task and most studies of ABMT have used this modified dot-probe approach: that is, participants are simultaneously presented with a disorder-relevant stimulus and a neutral stimulus on either side of a computer screen. Immediately after, a probe replaces one of the images and participants indicate the location of the probe as quickly as possible. When ABMT aims to reduce attention bias towards disorder-relevant stimuli the probe almost always replaces the neutral stimulus. When ABMT aims to increase attention bias to disorder-relevant stimuli, the probe almost always replaces the disorder-relevant stimuli. However, studies have shown that modified inhibitory control paradigms (e.g., the antisaccade task) may also alter responses in the context of appetitive stimuli, including food (1, 2).

In the present trial, ABMT was based on a modified version of the antisaccade task developed and piloted by Werthmann et al. (2). Training was completed on a personal laptop or desktop computer and lasted 10-15 minutes with breaks. Participants allocated to receive concurrent treatment (i.e., ABMT + real/sham tDCS) began ABMT five minutes after starting the stimulation so that ABMT and stimulation end approximately simultaneously.

The ABMT task consisted of 360 trials. Of these, 180 required participants to look towards low-calorie food cues, and 180 trials required participants to look away from high-calorie food cues. At the beginning of each trial, a black fixation point appeared for 100ms, followed by a red or blue fixation point (500ms). A blue point indicated that a pro-saccadic eye movement was required (i.e., look towards the food picture which appears after the fixation point), whereas a red point indicated that an anti-saccadic eye movement was required (i.e., direct the gaze away from the food picture which appears after the fixation point). Low-calorie cues were always preceded by a blue dot and high-calorie food cues were always preceded by a red dot. A blank screen was inserted for 200ms between the fixation point and the stimulus presentation. The pictorial stimulus (a high- or low-calorie food picture) then appeared on either the left or the right side of the screen for 500ms. Inter-trial interval was 1300ms. Trials were presented in a random order across three blocks, each including 120 trials. See Figure 1. for an example of a pro-saccade and anti-saccade stimulus presentation.

Pictorial stimuli were 30 low-calorie food and 30 high-calorie food pictures, which were visually matched for brightness, colour, and complexity, taken from Werthmann, Field (2). Each image was presented twice in each block, once on the left side of the screen and once on the right side of the screen (in a counterbalanced order), resulting in a total of 360 training trials (30 food stimuli + 30 non-food stimuli × 2 positions × 3 blocks).

In addition to directing their gaze towards or away from the stimulus presented, participants were instructed to press the arrow key which corresponds with the direction of their gaze. Response latencies were recorded to monitor accuracy and provide participants with feedback. For each block, number of correct responses was summed and presented as percentage score to the participant. Eye movements were not recorded during ABMT.

Figure 1. ABMT stimulus presentation.


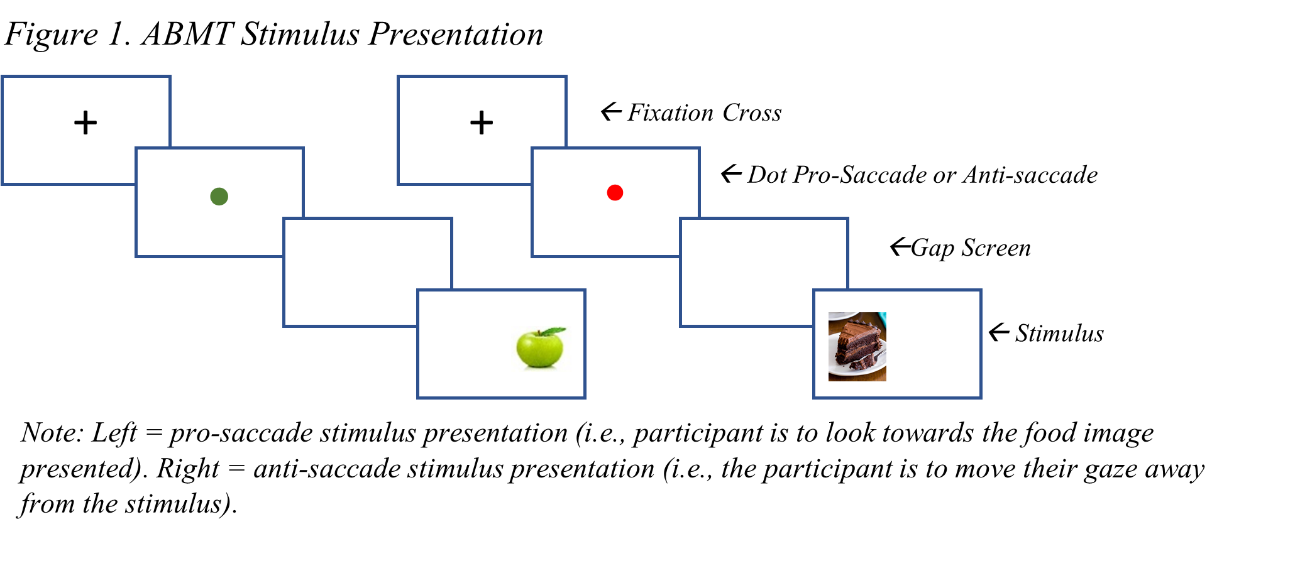


####

#### Self-Administered Transcranial Direct Current Stimulation (tDCS)

A specialist system designed for self-administration was used to deliver tDCS (the HDC Kit with MindCap™ by Newronika, Figure 2). This comprises of an easy to use, lay-person friendly stimulator, a programming device used by the researcher to securely set stimulation parameters, and a MindCap electrode placement system which ensures simple, safe, and reliable placement of the anode and cathode over the right and left DLPFC. The MindCap is a spandex cap which fits tightly like a swimming cap and is secured at the front (chin) and back (base of the scale) using Velcro. The system is 'one-size-fits all’ and resembles an EEG cap.

Stimulation was initiated by the participant and ran for 20 minutes, unless ended prematurely by the participant (e.g., due to discomfort). In the real tDCS condition, stimulation was delivered with the anode over the right dlPFC and the cathode over the left dlPFC at an intensity of 2mA for 20 minutes. In sham, participants set up electrodes in the same way and received active stimulation for 60 seconds at the start and end of the session.

Figure 2. Equipment for tDCS self-administration.


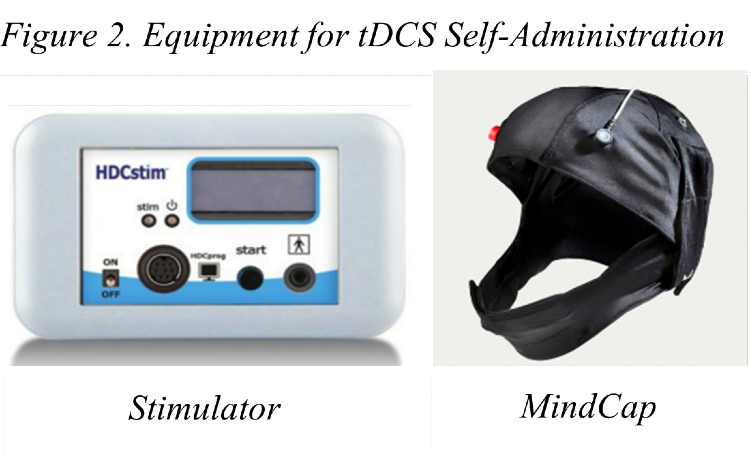


## Supplementary Feasibility Results

Table 1. Supplementary participant demographics

|  |  | **Whole Sample** | **Real tDCS+ABMT** | **Sham tDCS+ABMT** | **ABMT Only** | **Wait** |
| --- | --- | --- | --- | --- | --- | --- |
|  |  | *n* (%) | *n* (%) | *n* (%) | *n* (%) | *n* (%) |
| Highest Level of Education Achieved | | | | | | |
|  | University | 56 (68.29) | 16 (80.0) | 14 (70.00) | 13 (65.00) | 13 (68.42) |
|  | A or AS Levels | 14 (17.07) | 3 (15.0) | 2 (10.00) | 4 (20.00) | 2 (10.53) |
|  | GCSE | 7 (8.54) | 1 (5.0) | 4 (20.00) | 1 (5.00) | 1 (5.26) |
|  | Other tertiary | 5 (6.10) | 0 | 0 | 2 (10.00) | 3 (15.79) |
| Ethnicity | | | | | | |
|  | White | 75 (91.50) | 19 (95.0) | 19 (95.00) | 17 (85.00) | 17 (89.47) |
|  | Black | 3 (3.66) | 0 (0.00) | 0 (0.000 | 2 (10.00) | 1 (5.26) |
|  | Asian | 3 (3.66) | 0 (0.00) | 1 (5.00) | 1 (5.00) | 1 (5.26) |
|  | Latino | 1 (1.20) | 1 (5.0) | 0 | 0 | 0 |
| Marital Status | | | | | | |
|  | Single | 23 (28.05) | 5 (25.00) | 4 (20.00) | 5 (25.00) | 8 (42.11) |
|  | Dating | 10 (12.20) | 5 (25.00) | 1 (5.00) | 2 (10.00) | 2 (10.53) |
|  | Married* | 38 (46.34) | 7 (35.00) | 13 (65.00) | 11 (55.00) | 5 (26.32) |
|  | Divorced | 7 (8.54) | 1 (5.00) | 2 (10.00) | 2 (10.00) | 2 (10.53) |
|  | Other | 4 (4.88) | 1. (10.00) | 0 | 0 | 2 (10.53) |
| * Includes civil partnership and equivalent long-term relationships. SD, standard deviation; GCSE, General Certificate of Secondary Education; A, advanced; AS, Advanced Subsidiary; tDCS, transcranial direct current stimulation; ABMT, attention bias modification training. | | | | | | |

#### Blinding success

Participants did not distinguish real from sham tDCS at a rate better than chance [T1: 48.7% correct, ($x^{2}$(1) = 1.17, *p* = 0.533); T2: 41.0% correct, ($x^{2}$(1) = 1.21, p = 0.323)].

## Supplementary Clinical Results

Table 2. Mean change scores for clinical outcome measures.

|  | | **Real tDCS+ABMT** | | | **Sham tDCS+ABMT** | | | **ABMT Only** | | | **WL** | | |
| --- | --- | --- | --- | --- | --- | --- | --- | --- | --- | --- | --- | --- | --- |
|  | | ***N*** | $\bar{\boldsymbol{X}}$ | **SD** | ***n*** | $\bar{\boldsymbol{X}}$ | **SD** | ***n*** | $\bar{\boldsymbol{X}}$ | **SD** | ***n*** | $\bar{\boldsymbol{X}}$ | **SD** |
| ***Mean change from baseline (T0) to post-treatment (T1)*** | | | | | | | | | | | | | |
| BMI | | 20 | -0.48 | 0.59 | 19 | -0.36 | 0.56 | 20 | 0.14 | 0.49 | 17 | 0.01 | 0.14 |
| EDE-Q Global | | 20 | 3.00 | 0.83 | 19 | 3.40 | 1.03 | 20 | 3.62 | 0.99 | 17 | 4.07 | 1.14 |
| Monthly OBEs | | 20 | 10.95 | 5.93 | 19 | 13.26 | 15.23 | 20 | 12.45 | 8.81 | 17 | 18.29 | 7.88 |
| DASS-21 Total | | 20 | 31.80 | 16.86 | 19 | 35.37 | 20.59 | 20 | 34.10 | 18.69 | 17 | 40.71 | 23.54 |
|  | Depression | 20 | 13.50 | 8.31 | 19 | 13.47 | 8.87 | 20 | 14.00 | 10.24 | 17 | 14.82 | 10.91 |
|  | Anxiety | 20 | 5.50 | 4.58 | 19 | 7.89 | 7.84 | 20 | 7.50 | 6.15 | 17 | 9.06 | 8.72 |
|  | Stress | 20 | 12.80 | 7.15 | 19 | 14.00 | 6.32 | 20 | 13.30 | 6.88 | 17 | 16.47 | 8.41 |
| FCQ-Tr Total | | 20 | 49.40 | 14.46 | 19 | 50.95 | 15.57 | 20 | 51.75 | 14.92 | 17 | 63.00 | 8.16 |
| DERS | | 20 | 27.25 | 18.49 | 19 | 33.58 | 17.10 | 20 | 26.35 | 14.73 | 17 | 31.53 | 16.79 |
| CIA | | 20 | 1.22 | 0.51 | 19 | 1.35 | 0.46 | 20 | 1.32 | 0.56 | 17 | 1.47 | 0.63 |
| ***Mean change from baseline (T0) to follow-up (T2)*** | | | | | | | | | | | | | |
| BMI | | 20 | -1.28 | 0.48 | 19 | -0.52 | 0.45 | 19 | -0.07 | 0.52 | 17 | -.0.03 | 0.17 |
| EDE-Q Global | | 17 | 1.78 | 0.76 | 17 | 2.82 | 0.90 | 17 | 2.27 | 0.98 | 17 | 3.90 | 1.25 |
| Monthly OBEs | | 17 | 6.25 | 6.01 | 17 | 12.00 | 14.29 | 17 | 9.94 | 10.00 | 17 | 19.06 | 5.79 |
| DASS-21 Total | | 17 | 26.24 | 18.00 | 17 | 34.35 | 19.90 | 17 | 32.94 | 22.87 | 17 | 41.29 | 23.18 |
|  | Depression | 17 | 7.41 | 5.42 | 17 | 12.00 | 8.09 | 17 | 14.82 | 10.30 | 17 | 15.53 | 11.67 |
|  | Anxiety | 17 | 6.35 | 6.37 | 17 | 8.35 | 6.33 | 17 | 5.88 | 6.54 | 17 | 9.41 | 8.57 |
|  | Stress | 17 | 12.47 | 8.65 | 17 | 14.00 | 7.87 | 17 | 12.00 | 7.58 | 17 | 16.35 | 7.57 |
| FCQ-Tr Total | | 17 | 41.00 | 13.67 | 17 | 48.82 | 16.22 | 17 | 46.41 | 16.63 | 17 | 63.47 | 7.45 |
| DERS | | 17 | 22.47 | 14.86 | 17 | 28.50 | 15.26 | 17 | 28.18 | 12.01 | 17 | 32.59 | 7.41 |
| CIA | | 17 | 0.84 | 0.35 | 17 | 1.18 | 0.55 | 17 | 1.37 | 0.74 | 17 | 1.54 | 0.57 |
| Abbreviations (in alphabetical order): ABMT, attention bias modification training; BMI, body mass index; CIA, Clinical Impairment Assessment; DASS-21, Depression, Anxiety and Stress Scale – 21 items; DERS, Difficulties with Emotion Regulation Scale; EDE-Q; Eating Disorders Examination Questionnaire; FCQ-Tr, Food Craving Questionnaire-Trait reduced version; OBEs, objective binge eating episodes, tDCS, transcranial direct current stimulation; WL, wating list; $\bar{X}$, sample mean. | | | | | | | | | | | | | |

|  | Real tDCS+ABMT vs WL | | | | Sham tDCS+ABMT vs WL | | | | ABMT Only  vs WL | | | | Real tDCS+ABMT vs ABMT Only | | | | Sham tDCS+ABMT vs ABMT Only | | | | Real tDCS+ABMT vs Sham tDCS+ABMT | | | | |
| --- | --- | --- | --- | --- | --- | --- | --- | --- | --- | --- | --- | --- | --- | --- | --- | --- | --- | --- | --- | --- | --- | --- | --- | --- | --- |
|  |  | | 95% CI | |  | | 95% CI | |  | | 95% CI | |  | 95% CI | | |  | | 95% CI | |  | | | 95% CI | |
|  | *D* | Low | | High | *d* | Low | | High | | *d* | Low | High | *d* | | Low | High | *d* | Low | | High | | *d* | Low | | High |
| ***Change from baseline (T0) to post-treatment (T1)*** | | | | | | | | | | | | | | | | | | | | | | | | | |
| BMI | -1.11 | -1.78 | | -0.41 | -0.89 | -1.57 | | -0.21 | | 0.34 | -0.31 | 0.99 | -1.13 | | -1.80 | -0.44 | -0.95 | -1.61 | | -0.27 | | -0.20 | -0.83 | | 0.43 |
| EDE-Q Global | -1.92 | -2.70 | | -1.12 | -1.79 | -2.56 | | -1.00 | | -1.18 | -1.88 | -0.47 | -0.63 | | -1.26 | 0.01 | -0.05 | -0.67 | | 0.58 | | -0.73 | -1.37 | | -0.07 |
| Monthly OBEs | -0.90 | -1.58 | | -0.21 | -0.62 | -1.29 | | 0.05 | | -0.98 | -1.65 | -0.28 | 0.01 | | -0.63 | 0.62 | 0.35 | -0.29 | | 0.98 | | -0.33 | -0.96 | | 0.32 |
| DASS-21 Depression | -0.53 | -1.18 | | 0.14 | -0.10 | -0.75 | | 0.55 | | -0.53 | -1.18 | 0.14 | -0.06 | | -0.68 | 0.56 | 0.47 | -0.17 | | 1.10 | | -0.47 | -1.11 | | 0.17 |
| DASS-21 Anxiety | -0.63 | -1.29 | | 0.04 | -0.35 | -1.01 | | 0.31 | | -0.52 | -1.17 | 0.14 | -0.12 | | -0.74 | 0.50 | 0.31 | -0.33 | | 0.94 | | -0.47 | -1.11 | | 0.17 |
| DASS-21 Stress | -0.72 | -1.38 | | -0.05 | -0.39 | -1.05 | | 0.28 | | -0.89 | -1.56 | -0.21 | -0.08 | | -0.70 | 0.54 | 0.52 | -0.12 | | 1.16 | | -0.46 | -1.09 | | 0.18 |
| DASS-21 Total | -0.88 | -1.55 | | -0.20 | -0.45 | -1.11 | | 0.22 | | -0.96 | -1.64 | -0.27 | -0.14 | | -0.75 | 0.49 | 0.66 | 0.01 | | 1.30 | | -0.63 | -1.27 | | 0.01 |
| FCQ-Tr Total | -1.30 | -2.01 | | -0.58 | -0.85 | -1.53 | | -0.16 | | -1.17 | -1.86 | -0.46 | -0.14 | | -0.76 | 0.48 | 0.22 | -0.41 | | 0.85 | | -0.35 | -0.98 | | 0.28 |
| DERS | -1.47 | -2.19 | | -0.73 | 0.11 | -0.55 | | 0.76 | | -0.79 | -1.46 | -0.12 | -0.28 | | -0.90 | 0.35 | 0.84 | 0.17 | | 1.49 | | -1.39 | -2.09 | | -0.68 |
| CIA | -0.54 | -1.19 | | 0.13 | -0.12 | -0.78 | | 0.53 | | -0.85 | -1.52 | -0.17 | 0.13 | | -0.50 | 0.74 | 0.58 | -0.06 | | 1.22 | | -0.38 | -1.01 | | 0.26 |
| *Change from baseline (T0) to Follow-up (T2)* | | | | | | | | | | | | | | | | | | | | | | | | | |
| BMI | -1.21 | -1.90 | | -0.51 | -1.12 | -1.81 | | -0.42 | | 0.12 | -0.53 | 0.76 | -1.20 | | -1.88 | -0.51 | -0.98 | -1.65 | | -0.30 | | -0.66 | -1.30 | | -0.01 |
| EDE-Q Global | -2.45 | -3.34 | | -1.54 | -1.62 | -2.39 | | -0.83 | | -2.38 | -3.25 | -1.48 | -0.27 | | -0.94 | 0.41 | 0.87 | 0.16 | | 1.57 | | -1.09 | -1.80 | | -0.36 |
| Monthly OBEs | -1.33 | -2.08 | | -0.56 | -1.21 | -1.93 | | -0.46 | | -1.05 | -1.77 | -0.33 | -0.21 | | -0.90 | 0.47 | 0.32 | -0.36 | | 0.99 | | -0.58 | -1.28 | | 0.12 |
| DASS-21 Depression | -1.20 | -1.92 | | -0.46 | -0.19 | -0.87 | | 0.48 | | -0.62 | -1.31 | 0.07 | -0.86 | | -1.56 | -0.15 | 0.39 | -0.29 | | 1.07 | | -1.06 | -1.77 | | -0.33 |
| DASS-21 Anxiety | -0.67 | -1.35 | | 0.03 | -0.36 | -1.03 | | 0.32 | | -0.74 | -1.43 | -0.04 | 0.11 | | -0.56 | 0.78 | 0.52 | -0.17 | | 1.20 | | -0.42 | -1.10 | | 0.26 |
| DASS-21 Stress | -0.77 | -1.46 | | -0.07 | -0.39 | -1.07 | | 0.29 | | -1.05 | -1.76 | -0.32 | -0.01 | | -0.66 | 0.69 | 0.67 | -0.03 | | 1.35 | | -0.48 | -1.16 | | 0.20 |
| DASS-21 Total | -1.15 | -1.87 | | -0.41 | -0.41 | -1.09 | | 0.27 | | -1.18 | -1.90 | -0.44 | -0.41 | | -1.09 | 0.27 | 0.70 | 0.00 | | 1.38 | | -0.84 | -1.54 | | -0.13 |
| FCQ-Tr Total | -2.06 | -2.89 | | -1.21 | -0.93 | -1.64 | | -0.22 | | -1.26 | -2.00 | -0.52 | -0.36 | | -1.03 | 0.32 | 0.27 | -0.41 | | 0.94 | | -0.66 | -1.35 | | 0.03 |
| DERS | -1.56 | -2.32 | | -0.77 | -0.43 | -1.11 | | 0.27 | | -0.68 | -1.37 | 0.02 | -0.67 | | -1.35 | 0.03 | 0.22 | -0.47 | | 0.90 | | -0.91 | -1.62 | | -0.18 |
| CIA | -1.46 | -2.21 | | -0.69 | -0.67 | -1.36 | | 0.02 | | -0.69 | -1.38 | 0.01 | -0.66 | | -1.35 | 0.03 | 0.07 | -0.60 | | 0.74 | | -0.77 | -1.46 | | -0.06 |
| Change scores = post-treatment/follow-up scores minus baseline scores. Positive *d* values indicate that M_1_ was higher than M_2_, negative *d* values indicate that M_1_ was lower than M_2_. Abbreviations (in alphabetical order): ABMT, attention bias modification training; BMI, body mass index; CI, confidence interval; CIA, Clinical Impairment Assessment; *d,* effect size (Cohen’s *d);* DASS-21, Depression, Anxiety and Stress Scale – 21 items; DERS, Difficulties with Emotion Regulation Scale; EDE-Q; Eating Disorders Examination Questionnaire; FCQ-Tr, Food Craving Questionnaire-Trait reduced version; *n,* number of observations, OBEs, objective binge eating episodes; tDCS, transcranial direct current stimulation; WL, waiting list. | | | | | | | | | | | | | | | | | | | | | | | | | |

Table 3. Between-group effect sizes for change scores for clinical outcome measures.

*BMI*

At T2, the real tDCS+ABMT group reported a mean weight loss since T0 equivalent to a 1.28-point reduction in BMI (SD = 0.48). In sham tDCS+ABMT, at T2, BMI was reduced by 0.52 points (SD = 0.56). BMI change from T0 to T2 was negligible for ABMT only ($\mathrm{mean}_{\Delta}$ = -0.07 points, SD = 0.52) and WL groups ($\mathrm{mean}_{\Delta}$ = -0.03, SD = 0.17).

When comparing real tDCS+ABMT and sham tDCS+ABMT with WL, effect sizes for change scores were large and favoured intervention groups. Similarly, when comparing real tDCS+ABMT and sham tDCS+ABMT with ABMT only, effect sizes for BMI change were large and favoured the combined real/sham interventions. The effect size for BMI change score comparing real tDCS+ABMT with sham tDCS+ABMT was small and favoured real tDCS+ABMT.

Supplementary Figure 3**.** BMI from baseline to follow-up (error bars show 95% CI)


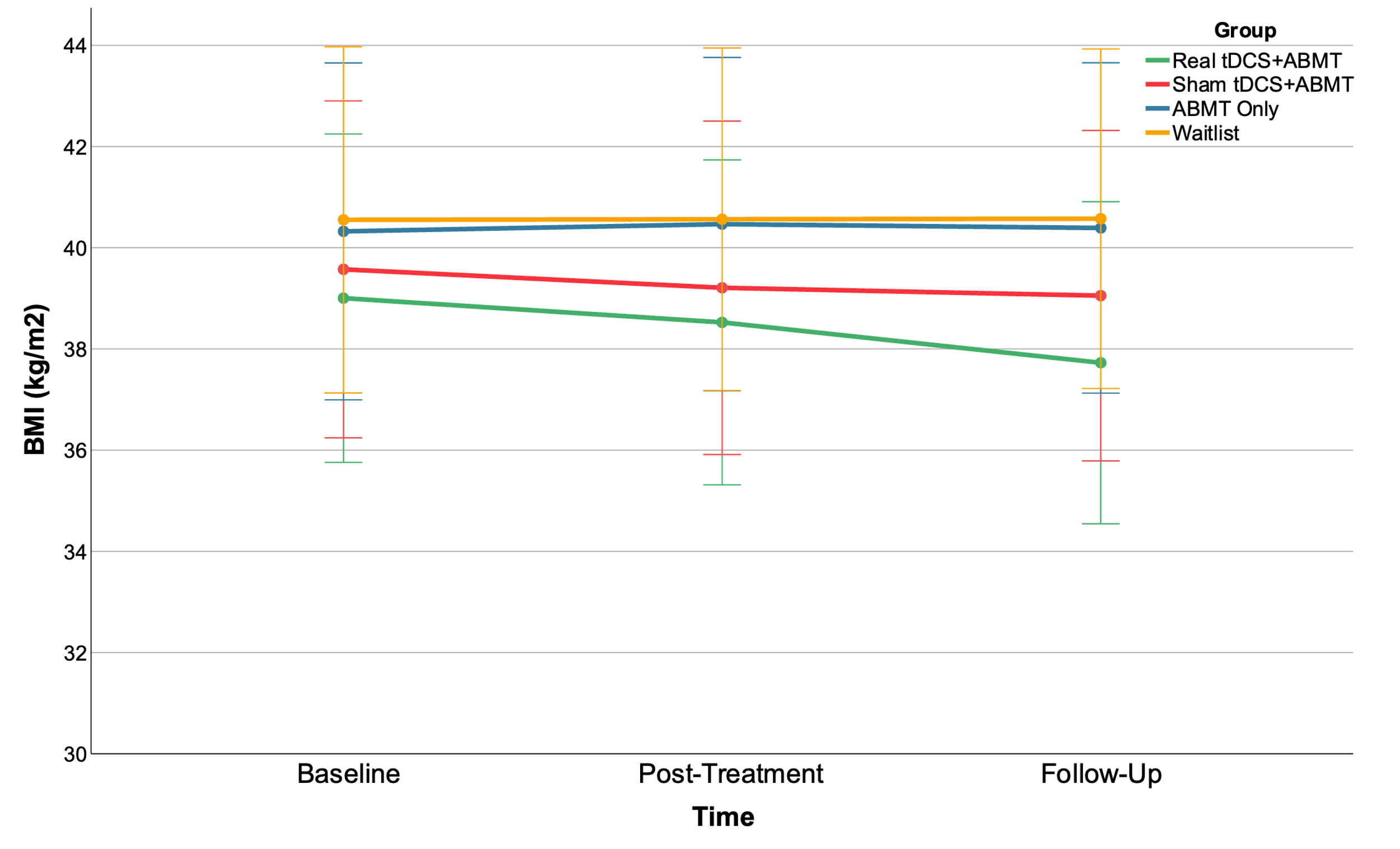


### ED psychopathology

By T2, ED symptom severity was reduced in all intervention groups, as demonstrated by a decrease in the EDE-Q global score over time, and large effect sizes for change scores relative to WL. When comparing real tDCS+ABMT with sham tDCS+ABMT, the effect size for change score was large and favoured real tDCS+ABMT. The effect size for change score comparing real tDCS+ABMT with ABMT was small and favoured the real tDCS+ABMT group. When comparing sham tDCS+ABMT with ABMT only, a large effect size for change score favoured ABMT only.

Supplementary Figure 4. Eating disorder symptoms (EDE-Q global score) from baseline to follow-up (error bars show 95% CI)


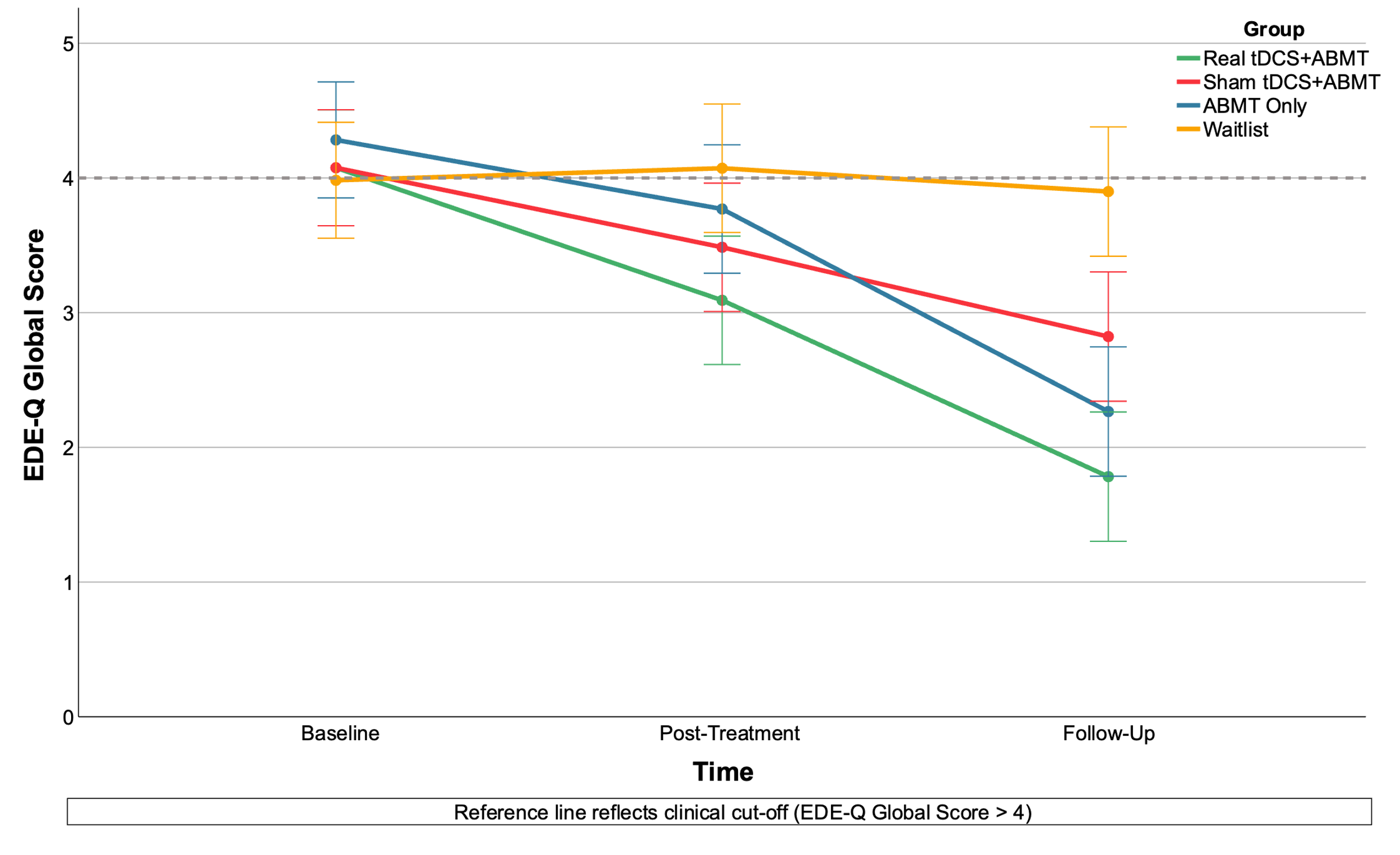


### Craving for food

Trait craving for food, as measured by the FCQ-Tr, was reduced in each intervention by T2, and large effect sizes for change scores relative to WL were observed. When comparing real tDCS with other intervention groups, effect sizes for change scores to T2 were small-to-moderate and favoured the real tDCS+ABMT group. When comparing sham tDCS+ABMT with ABMT only, the effect size for T2 change score was negligible.

Supplementary Figure 5. Trait craving for food (FCQ-T total score) from baseline to follow-up (error bars show 95% CI).


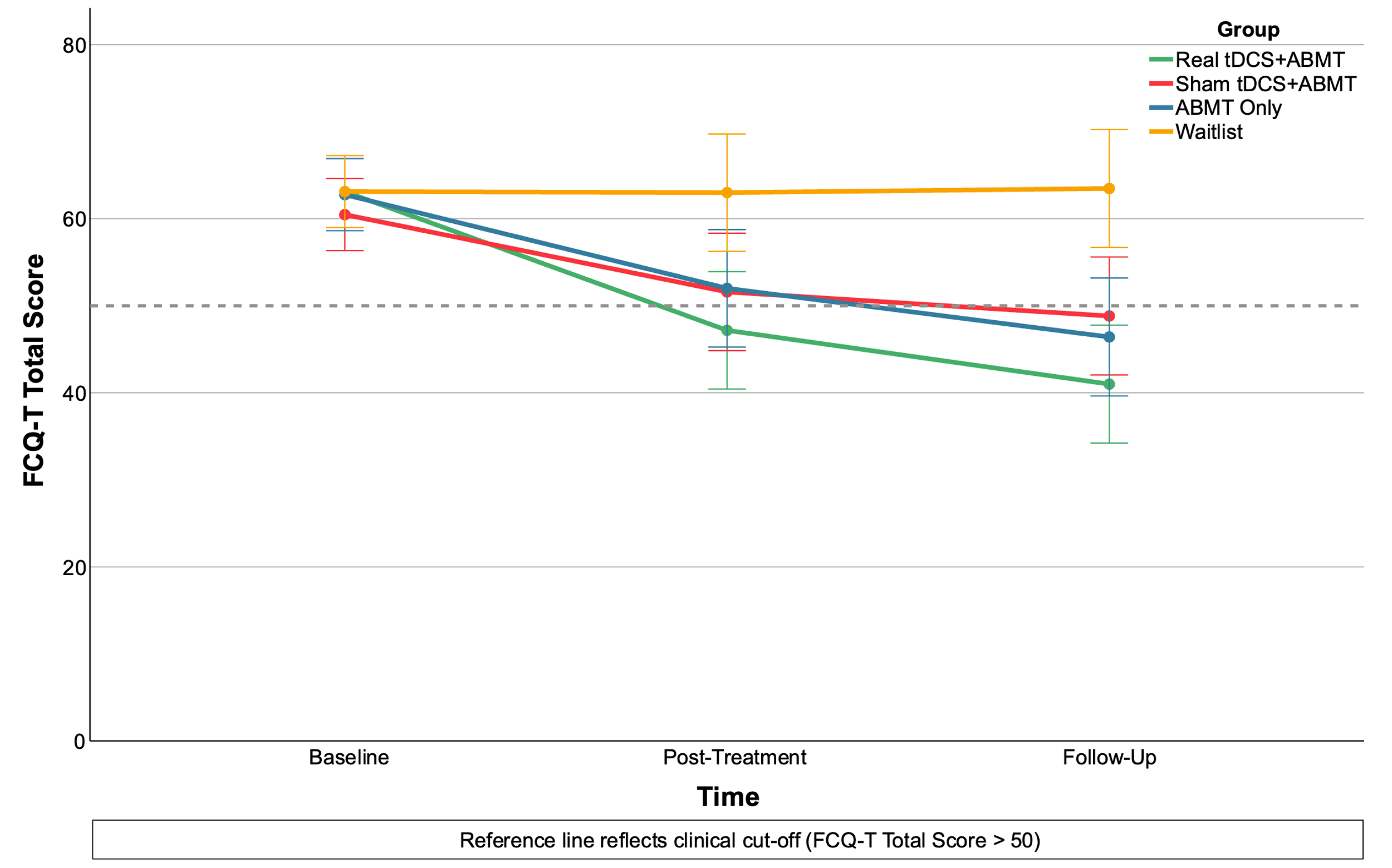


##### General psychopathology

By T2, DASS-21 total scores were reduced in all intervention groups (Supplementary Figure 4), and when comparing intervention groups to WL, moderate-to-large effect sizes for change scores favoured the intervention groups. When comparing real tDCS+ABMT with sham tDCS+ABMT and ABMT only, moderate-to-large effect sizes for change scores favoured the real tDCS+ABMT group. A moderate effect size for change score favoured ABMT only, as opposed to sham tDCS+ABMT.

Supplementary Figure 6. General psychopathology (DASS-21 total score) from baseline to follow-up (error bars show 95% CI).


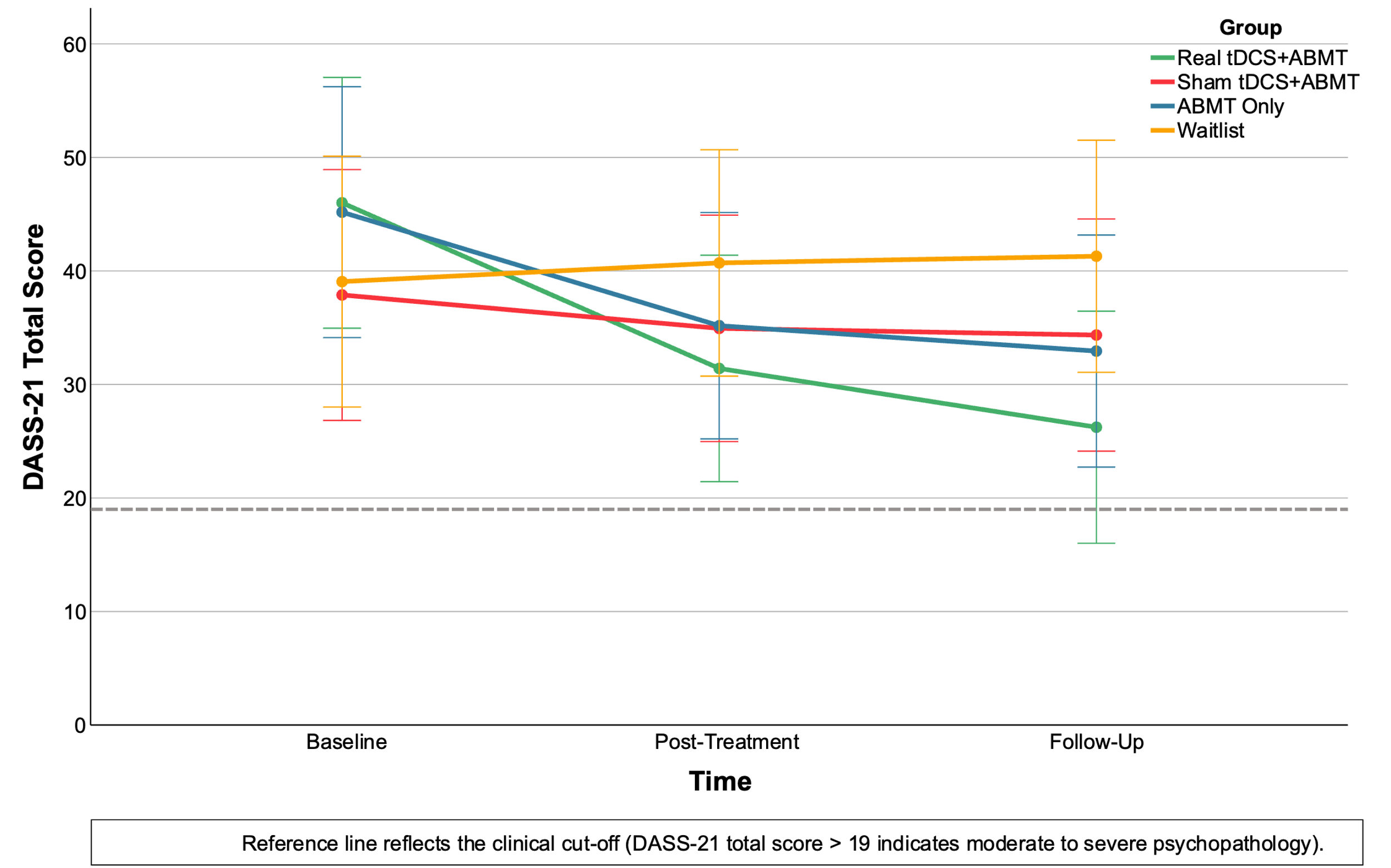


### Difficulties with emotion regulation

With respect to change in self-reported difficulties with emotion regulation, as measured by the DERS, moderate-to-large effect sizes for change scores from baseline to follow-up favoured the real tDCS+ABMT group, as opposed to WL control (real tDCS+ABMT: *d* = -1.15, 95% CI = -1.87, -0.41), sham tDCS+ABMT (*d* = *-*0.91, 95% CI = -1.62, -018), and ABMT only (*d* = -0.67, 95% CI = -1.35, 0.03). For sham tDCS+ABMT and ABMT only, effect sizes for DERS change scores to follow-up were moderate and favoured the intervention groups, as opposed to WL (sham tDCS+ABMT: *d* = -0.43, 95% CI = -1.11, 0.27; ABMT only: *d =* -0.68, 95% CI = -1.37, 0.02). Small effect sizes for DERS change scores favoured the sham tDCS+ABMT group, as opposed to ABMT only (*d* = -0.22, -0.47, -0.90).

Supplementary Figure 7. Difficulties with emotion regulation (DERS) from baseline to follow-up (error bars show 95% CI).


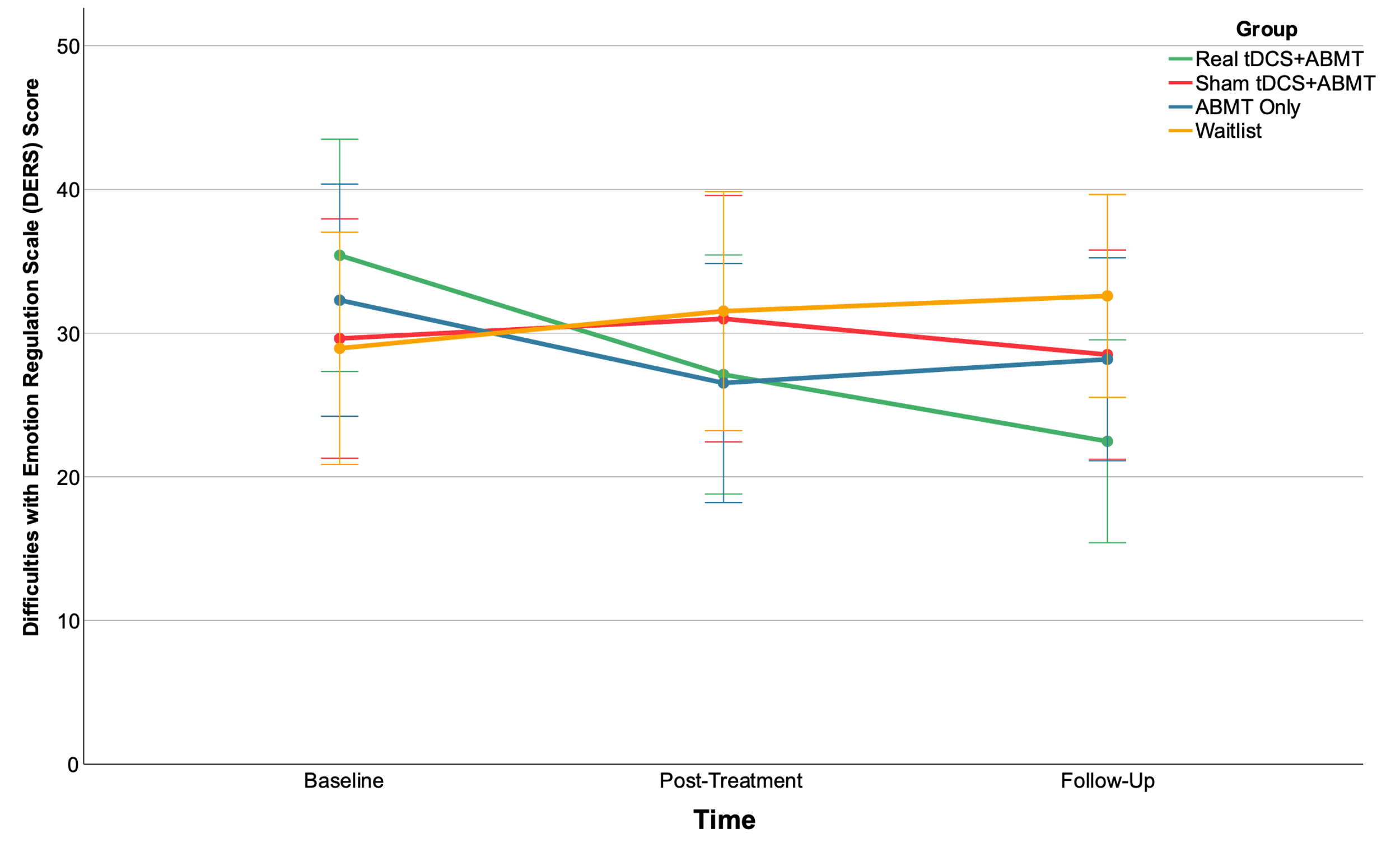


### ED related clinical impairment.

Change to quality of life was examined using the CIA. Relative to WL, moderate-to-large effect sizes for change scores favoured intervention groups (real tDCS+ABMT: *d*= -1.46, 95% CI = -2.21, -0.69; sham tDCS+ABMT: *d*= -0.67, 95% CI = -1.36, 0.02; ABMT only: *d*= -0.69, 95% CI = -1.38, 0.01). Similarly, moderate effect sizes favoured the real tDCS+ABMT group, as opposed to ABMT only (*d*= -0.66, 95% CI = -1.35, 0.03) and sham tDCS+ABMT (*d* = 0.77, 95% CI = -1.46, -0.06). No difference in change scores from baseline to follow was observed for sham tDCS+ABMT relative to ABMT only (*d* = 0.03, 95% CI = 0.07, -0.60).

Supplementary Figure 8. ED related impairment (CIA Score) from baseline to follow-up (error bars show 95% CI).


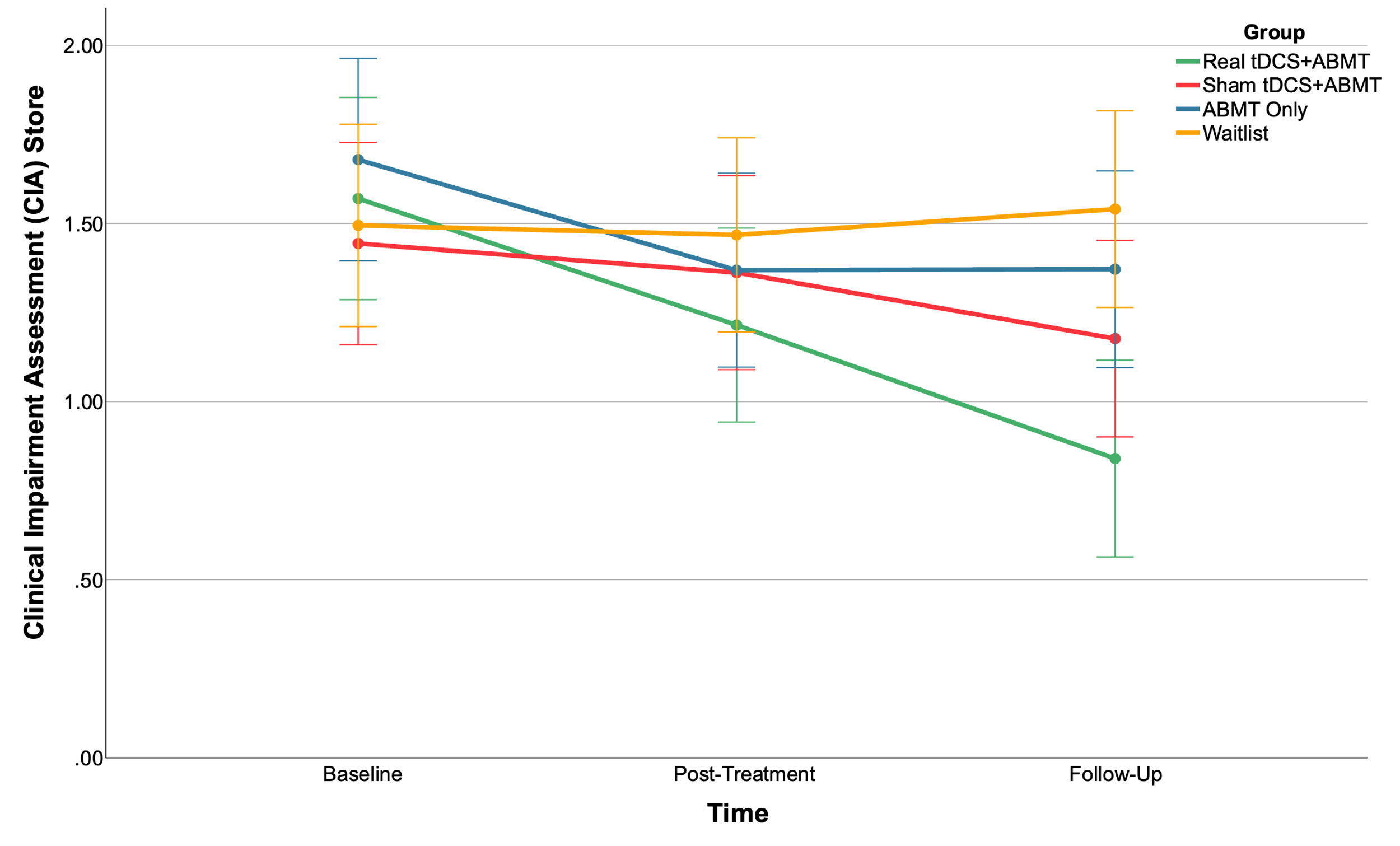


### Sample Size Estimation for a future trial

Based on TANDEM findings, a conservative estimate of the sample size needed to detect a difference between groups would use the smallest effect size for OBE change score. This was observed when comparing tDCS with ABMT and ABMT only (d = -0.21). Based on this approach, a future large scale RCT with 4 arms (real tDCS with ABMT, sham tDCS with ABMT, ABMT only, and WL), a significance criterion of α = .05 and power = .80, the minimum sample size needed to detect a difference between the real and sham groups with this effect size is N = 676 (169 participants per group). Assuming drop-out rates were low, as in this feasibility trial (3.6% drop out rate), a total of 703 participants would need to be recruited.

Given the large sample size needed to use the same study design, future trials could consider an RCT with two arms (real tDCS with ABMT and sham tDCS with ABMT) or three arms (as previously plus WL). Using the effect size for OBE change score comparing real tDCS+ABMT with sham tDCS+ABMT, a two-arm trial with a significance criterion of α = .05, power = .80 and accounting for 3.6% drop out rate, the minimum sample size needed to detect a difference between the real and sham groups with this effect size is N = 70 (35 participants per group). For a three arm RCT, a minimum of 84 participants would be needed (28 per group).

## References

1. Giel KE, Schag K, Plewnia C, Zipfel S. Antisaccadic training to improve impulsivity in binge eating disorder. European Eating Disorders Review. 2013;21(6):488-92.

2. Werthmann J, Field M, Roefs A, Nederkoorn C, Jansen A. Attention bias for chocolate increases chocolate consumption: An attention bias modification study. Journal of Behavior Therapy & Experimental Psychiatry. 2014;45(1):136-43.
